# Multiple Gestation after Elective Single-Embryo Transfer: A State-of-the-Art Review of Literature and Suggested Mechanisms

**DOI:** 10.1155/2024/2686128

**Published:** 2024-01-16

**Authors:** Mokhamad Zhaffal, Rania Al Jafari, Anastasia Salame

**Affiliations:** ^1^Obstetrics and Gynaecology Department, UAE University, Al Ain, UAE; ^2^Fakih Fertility Center, Al Ain, UAE; ^3^IVF Department, Fakih Fertility Center, Al Ain, UAE

## Abstract

**Background:**

Elective single-embryo transfers are being increasingly used to curb the increase in multiple gestation rates. However, it has been documented that single-embryo transfers could still result in twins and on rarer occasions in triplet pregnancies. *Main Body*. A literature review was done to highlight the possible mechanisms leading to embryonic splitting. In this review, the incidence of zygotic splitting was addressed and the notion of chorionicity was explained. Risk factors for zygotic splitting and suggested mechanisms for both twin and higher order pregnancies were suggested and discussed.

**Conclusion:**

The hypotheses that we have so far remain unproven due to the rarity of zygotic splitting as well as the ethicolegal considerations of human embryo research. The presence of such incidents necessitates extensive counselling of the couple.

## 1. Introduction

Multiple gestations are the most frequent complications associated with assisted reproductive techniques (ART). To decrease the rate of twin and higher order multiple gestations, the elective single-embryo transfer (eSET) is currently encouraged. However, blastocyst stage SET was still found to be associated with multiple gestations. The present data revealed that the frequency of embryo splitting post-eSET is 1.36% with the rate of monozygotic twin and triplet pregnancies being 0.9-3.1% and 0.048%, respectively [1, 2]. However, dizygotic twin and triplet pregnancies can also be encountered post-eSET [3]. Both monozygotic and dizygotic pregnancies can occur in the setting of fresh embryo transfers, in natural or modified natural frozen embryo transfers (FET), and rarely in medicated FET cycles [4, 5]. Such events challenge the twinning dogma proposed by Corner [6].

## 2. Incidence of Zygotic Splitting after SET

Single-embryo transfer is becoming the most favored method of ART conclusion in recent years as it decreased the complications associated with multiple gestations. In Japan, for example, up to 80% of cycles account for SET [7]. However, multiple gestations in the form of mono- or dizygotic twinning are still encountered. The classic definition of when one embryo undergoes fission into 2 or more genetically identical embryos is called monozygotic splitting, while when 2 different embryos implant, a dizygotic pregnancy ensures. According to Ikemoto et al., the rate of multiple gestation after eSET is 1.6% with the frequency of twin and triplet pregnancies being 1.56% and 0.04%, respectively. The frequency of zygotic splitting post-SET was estimated to be 1.36% [2]. The findings of monochorionic and multiple chorionic pregnancies in blastocyst eSET confirmed the fact that embryo splitting took place after the transfer. However, not all pregnancies are monozygotic. As per Osianlis et al., the calculated dizygotic rate in their paper was 0.5% with an overall Di-Di birth rate of 1%. Based on these numbers, they concluded that 50% of the multizygotic pregnancies are due to actual embryo splitting while the other 50% could be explained by concomitant natural conception at the same time of the ART conception. On another level, the Japanese ART national registry database along with a survey done by Yamashita et al. documented 122 triplet pregnancies, of which 46 were single gestational sac pregnancies, 18 were double gestational sac pregnancies, and 59 were with 3 gestational sacs. It is worth mentioning that the trichorionic pregnancies had zero fetuses in 9 cases, 1 fetus in 12 cases, 2 fetuses in 9 cases, and three fetuses in 29 cases. One quadruplet case was also documented [8].

## 3. Chorionicity

Chorionicity refers to the placenta the origin of which can be determined accurately. Zygosity on the other hand, which is the origin of the fetus, can be predicted in half of the cases as multiple gestation pregnancies can originate from one or multiple embryos especially when the sex of the babies is discordant. Given this fact, same-sex twins or triplets could be true monozygotic or dizygotic in origin. The only way to accurately diagnose the zygosity is to do DNA fingerprinting which is expensive and thus not performed in daily practice [3]. In contrast, monochorionic multiple gestations are always monozygotic. Originally, it was thought that the earlier the embryonic division, the more separate and independent the fetuses were. In other words, cleavage stage divisions were believed to result in dichorionic diamniotic pregnancies while blastocyst stage divisions resulted in monochorionic monoamniotic pregnancies.

According to Konno et al., dichorionic pregnancies were found to be more common with ART [9]. As such, we can conclude that SET can result in monozygotic (monochorionic and multichorionic) as well as dizygotic pregnancies (multichorionic pregnancies).

## 4. Risk Factors

Naturally occurring twinning, especially the dizygotic form, is believed to be linked to a genetic predisposition most commonly located on chromosome 3 [10, 11]. Some ethnicities were found to be more predisposed to dizygotic twinning where the rate reached 50/1000 in Nigeria [12]. This contrasts with the naturally occurring monozygotic twinning which was found to be nonaffected by the ethnicity or the genetic makeup of the couple [12, 13]. ART on the other hand has increased the incidence of monozygotic twining. It has been shown that the patient's young age might predispose to zygotic twinning while unexplained infertility was found to be protective [2]. It was proposed that ART-associated embryo manipulations such as FET per se, blastocyst culture, and assisted hatching could be risk factors for zygotic splitting while the zona manipulation of the oocyte in the form of intracytoplasmic sperm insemination (ICSI) was not [2, 3, 14]. Interestingly, there was no difference in the splitting rate neither between the cleavage stage and the blastocyst stage transfers nor between fresh and frozen embryo transfer cycles [3]. Another risk factor for splitting is a lower inner cell mass (ICM) grading of B or C. It is thought that loose intercellular connections may induce the ICM fission [15]. This has been documented through the time-lapse imaging [16]. The quality of the culture media is also thought to stimulate zygotic splitting. An increase in the free radicals' concentration due to increased glucose concentration in the culture media used for prolonged culture could lead to ICM splitting at the site of glucose-induced apoptosis of certain regions of the ICM. The new sequential culture systems with antioxidant activity might explain the lack of increase of the rate of embryo splitting despite the major increase in the number of IVF cycles and embryo transfers worldwide. When coupled with the improvement in the embryologists training and experience, the rate of splitting associated with a blastocyst transfer has been found to decrease [17]. The OR for embryo splitting decreased from 2.2 to 1.7 when comparing the periods of 2007 to 2010 and 2010 to 2014 [2]. Embryo biopsy on the other hand was not found to increase the risk of embryo splitting contrary to what was believed before [18].

## 5. Suggested Mechanisms of Division

It has been shown that blastomeres from a 4-cell stage embryo can develop into an ICM and trophectoderm; hence, any division after this stage could give rise to 2 or more embryos with an implantation potential [19]. Of the suggested mechanisms, abnormal cellular axis formation and cytoplasm folding in the secondary oocyte prior to fertilization or during the actual fertilization lead to duplication. It is speculated that gonadotropin stimulation might disrupt the fine balance and gradients of signalling molecules affecting the polarity of the oocyte. This is thought to lead to the formation of 2 cells referred to as daughter cells or tertiary oocytes that could be fertilized. This is speculated to be caused by the displacement of the meiotic spindle due to oocyte aging postovulation. This disruption might lead to the duplication of the axes and formation of 2 embryos upon fertilization or the fission of the ICM into 2 at the blastocyst stage [13]. This theory would be replaced later on by the formation of 2 zygotes postfertilization of the secondary oocyte and not 2 blastomeres [20]. Other studies advocated the fission to happen closer to the cleavage stage, and thus, the sequence of events happening during hatching would then explain the type of the twin gestation. If both blastocysts were released at the same time, then the resultant pregnancy would be a dichorionic diamniotic twin gestation. If on the other hand the blastocysts fused with the conservation of 2 separate ICMs prior to hatching, then monochorionic diamniotic twins would appear. If complete fusion of the trophectoderm and the ICM happened, then monochorionic monoamniotic twins would be created [21]. Another suggestion was that the ICM would split due to mechanical compression during hatching through the manipulated zona pellucida of the embryo which is also referred to as atypical hatching [22]. This atypical hatching is referred to as 8-shaped hatching, which usually takes place when the embryo is squeezing out through the hatch of the hardened zona pellucida due to prolonged culture to blastocyst stage and in cryopreserved-thawed blastocysts especially with the application of the day 3 prehatching protocol [23, 24]. It is speculated that this phenomenon might also be the culprit for the monozygotic triplet gestations that have been documented post-SET [16]. Another possible explanation to dual or even more ICM is the nature of human blastomere plasticity. Studies have shown that isolated trophectoderm cells when cultivated could give rise to a whole new embryo with an implantation potential [25]. As such, if a blastomere gets separated from the trophectoderm into the blastocele due to low-grade compaction of the trophectoderm, this blastomere could give rise to an ICM. Theoretically, each ICM should give rise to a separate fetus with the surrounding amnion while the chorion develops during the implantation. The mechanism of chorionic differentiation between mono and higher order chorionicity in monozygotic pregnancies is still unknown.

## 6. Triplets: Possible Explanation

The explanation of embryo splitting into three is challenging since triplets after SET is a very rare event. What is known so far is that to have implantation, an embryo with an intact ICM should be present. The number of the ICM that the embryo has will define the number of fetuses that will be seen on the pregnancy ultrasound. The chorionicity of the pregnancy will depend on the number of zygotes present at the time of implantation. In theory, the chorion should rise from trophectoderm cells; thus, it would be logical to consider that the higher the order of the chorionicity, the higher the number of separate embryos available for implantation. In the setting of monochorionic triplets, it is believed that the blastocyst harbors three distinct ICMs. The mechanism of their creation might be similar to the ones suggested for the monochorionic twin gestation. The trigger factor for the splitting into 3 and not into 2 is still unknown. In the setting of the multichorionic triplets, a suggested explanation might be the complete division of the hatching embryos resulting in 3 and not only 1 fully hatched embryo. Another possible explanation for this is if the origin of the sister ICMs is a trophectoderm blastomere. Since the implantation potential of reconstructed embryos cannot be tested at this point due to ethical reasons, one can only postulate that such cellular plasticity might confer to the newly formed ICM the whole genetic makeup necessary for a successful implantation and healthy fetal development.

The question that arises here is that whether the embryo initially had multiple ICM followed by trophectoderm splitting upon hatching or the splitting of the ICM took place during hatching due to the mechanical pressure exerted by the hardened zona pellucida. The latter might explain the high incidence of blighted ova in triplet pregnancies. Due to the abnormal cell division in the embryo(s) as well as increased cellular stress, the ICM fails to continue its division resulting in a blighted ovum.

## 7. Conclusion

Zygotic splitting is a well-described event in ART, yet the complete mechanism of these events is not completely elucidated. The hypotheses that we have so far remain unproven due to the rarity of zygotic splitting as well as the ethicolegal considerations of human embryo research. The presence of such incidents necessitates extensive counselling of couples undergoing SET.

## Abbreviations

ART: Assisted reproductive techniques
eSET: Elective single-embryo transfer
FET: Frozen embryo transfers
ICM: Inner cell mass
ICSI: Intracytoplasmic sperm injection.
